# Topology Optimization of Metal and Carbon Fiber Reinforced Plastic (CFRP) Laminated Battery-Hanging Structure

**DOI:** 10.3390/polym12112495

**Published:** 2020-10-27

**Authors:** Jiaju Chen, Yanan Xu, Yunkai Gao

**Affiliations:** 1 School of Automotive Studies, Tongji University, Shanghai 201804, China; chenjj@tongji.edu.cn (J.C.); xuyanan0416@163.com (Y.X.); 2 Shanghai Key Lab of Vehicle Aerodynamics and Vehicle Thermal Management Systems, Tongji University, Shanghai 201804, China

**Keywords:** topology optimization, carbon fiber reinforced plastic (CFRP), discrete material and thickness topology optimization (DMTO), electric vehicle

## Abstract

This study addressed the topology optimization of a carbon fiber reinforced plastic (CFRP) laminated battery-hanging structure of an electric vehicle. To accommodate parameterization for thickness and orientation of CFRP materials, the discrete material and thickness optimization (DMTO) technique was adopted. To include metal material as a reinforcement structure into the optimization simultaneously, the DMTO technique was extended here to achieve concurrent optimization of CFRP thickness topology, CFRP orientation selection and the topology of the metal reinforcement plate. Manufacturing constraints were applied, including suppressing intermediate void across the thickness direction of the laminate, contiguity constraint and the symmetrical layers. Sensitivities of the objective function were derived with respect to design variables. To calculate analytical sensitivities, finite element analysis was conducted and strain vectors were exported from a commercial software (ABAQUS) into a mathematical analysis tool (MATLAB). The design objective was to minimize the local displacement subject to the constraints of manufacturing and mass fraction. The mechanical performance of the optimized CFRP structure was compared with the original steel structure. To validate the optimization results, a prototype of the CFRP battery-hanging structure was fabricated and experimental testing was conducted. The results show that the total mass of the CFRP battery-hanging structure was reduced by 34.3% when compared with the steel one, while the mechanical property was improved by 25.3%.

## 1. Introduction

The transportation sector of the modern world mainly relies on fossil fuel. The use large amounts of fossil fuel is responsible for global warming, air pollution and ozone layer depletion. Besides, excessive usage of fossil fuel in vehicles is causing underground petroleum resources to dwindle. Environmental protection, lowering of tailpipe emissions and energy efficiency are among the most important goals of traffic policies, as transportation contributes to nearly one-third of greenhouse gas emissions [1,2]. In the automotive industry, environmental protection by reducing emissions has recently been attracting increased attention. In order to achieve this target, a transition towards electrification of urban mobility and transport is imminent. The immediate need for electric vehicles is driven by the forecasted shortage of crude oil and the necessity to reduce greenhouse gas emissions. Another critical factor in addressing this issue is the realization of lightweight automobile structures [3], so weight optimization of electrical vehicles is urgently demanded. The applications of novel materials, optimized topology structures and advanced processing technologies are effective ways to reduce the overall weight of automobiles. Compared with conventional metal materials, carbon fiber reinforced plastic (CFRP) materials have the advantage of high specific properties (high elastic modulus and high strength combined with low density); therefore, they are attractive for their applications in not only the automotive industry but also the aerospace and construction industries.

In fact, there are many studies underway to replace metal components with CFRP materials in vehicle structures. Wu et al. [4] addressed the design of ply orientation for a CFRP vehicle door in a general-purpose commercial finite element code (ABAQUS) and mathematical analysis tool (MATLAB); thereafter, Wu et al. [5] developed a discrete topology optimization procedure for the simultaneous design of ply orientation and thickness for a CFRP-laminated engine hood. An explicit parameterization for casing constraints was used to suppress intermediate void across the thickness of laminates. Lee et al. [6] proposed a set of rules to design automotive parts with equivalent bending stiffness through quasi-isotropic lay-up methods and genetic algorithms (GAS). CFRP B-pillar reinforcement was fabricated and investigated by the drop tower test. To reduce the weight compared to conventional structures, Lee et al. [7] applied a foam-cored CFRP sandwich composite to an automotive rear spoiler, and the foam-cored CFRP sandwich composite spoiler was compared with the conventional counterpart in terms of mechanical behavior through finite element analysis.

Optimization of CFRP structures is a good way to maximize the advantages of composite materials. Optimization of CFRP materials mainly includes two aspects, i.e., orientation optimization and thickness optimization. Structural performance may be completely different if different fiber orientations are selected due to anisotropic properties of CFRP materials [8]. Actually, it provides opportunities to achieve the desired performance of the composite structures by selecting appropriate fiber orientations in each layer. As mentioned above, CFRP structures usually weigh less than conventional metal structures and have the same or even better mechanical performance because of the high elastic modulus, high strength and low density of CFRP materials. Moreover, thickness optimization or topology optimization will further reduce the weight of structures through reasonable material distribution. When it comes to design of ply orientation or stacking sequence without taking layer thickness as a design variable, the majority of existing studies mainly use heuristic optimization methods [9,10,11,12,13,14,15,16,17]. The advantages of heuristic optimization reside in its possibility of convergence to a global optimum and conventional sensitivity analysis being unnecessary. However, it is often difficult to perform heuristic optimization when tackling large-scale design problems because of the high computational cost.

To solve the problems of computational burden associated with heuristic optimization, various gradient-based topology optimization methods have been proposed to optimize FRP composite structures, such as the shape functions with penalization (SFP) method proposed by Bruyneel [18], the bi-value coding parameterization (BCP) approach introduced by Tao et al. [19], the discrete material optimization (DMO) method [20] and the discrete material and thickness optimization (DMTO) [21] method proposed by Stegmann and Lund.

In automotive industry, CFRP structures need to be connected with other components. Therefore, numerous studies have been carried out for analysis of CFRP-to-metal joints [22,23,24,25], and metal materials are usually treated as reinforcements. However, the study of hybrid joints is outside the scope of this work. The novelty of this study includes the following points: (a) this study aimed to conduct concurrent topology optimization of a CFRP-laminated and metal reinforcement plate of a battery-hanging structure; (b) fiber ply orientation, ply thickness and metal plate thickness were regarded as design variables simultaneously, allowing the design room for CFRP optimization to be expanded and signifying an opportunity to achieve better performance of the structures; and (c) a prototype of the CFRP battery-hanging structure was fabricated and experimental testing was conducted. The design objective was to minimize the local displacement subject to the volume fraction and manufacturing constraints. Design sensitivities were derived with respect to design variables, and the method of moving asymptotes (MMA) was employed for driving optimization.

The remainder of this paper is organized as follows: The DMTO method is first depicted in Section 2, and the manufacturing constraints, filtering technique and the design sensitivities are discussed in the same section. Section 3 illustrates the optimization of a CFRP battery-hanging structure, followed by the manufacturing and experimental validation of the optimized CFRP structure in Section 4. Finally, conclusions are presented in Section 5.

## 2. Methodology

### 2.1. Discrete Material Topology Optimization

In this study, discrete material topology optimization (DMTO) approach was employed to parameterize different ply orientations ($x_{lc}$) and thickness ($\rho_{el}$) variables, which are given respectively as

(1) $$x_{lc}=\{\begin{array}{c} 1,\;if\;candidate\;orientation\;c\;is\;chosen\;in\;layer\;l\\ 0,\;otherwise \end{array}\;\rho_{el}=\{\begin{array}{c} 1,\;if\;there\;is\;a\;material\;in\;layer\;l\;for\;element\;e\\ 0,\;otherwise \end{array}$$

where $l=1,2,\ldots ,N^{l}$ stands for the ply number in each element ($N^{l}$ is the total number of plies); $c=1,2,\ldots ,N^{c}$ refers to the number of candidate materials ($N^{c}$ is the total number of candidate materials); and $e=1,2,\ldots ,N^{e}$ denotes to the number of elements ($N^{e}$ is the total number of elements).

Based on the definition of the above candidate material variables, the constitutive material matrix in layer l of element e can be expressed as

(2) $$E_{el}=E_{0}+\rho_{el}\overset{Nc}{\underset{c=1}{\sum }}x_{lc}\left(E_{c}-E_{0}\right)$$

where $E_{0}$ is the constitutive matrix of the artificial material to avoid singularity and $E_{c}$ represents the constitutive matrix of candidate material c. Here, $x_{lc}$ can also be regarded as a topological variable. When $x_{lc}=0$, then candidate material c will not be applied to layer *l*. In order to ensure that only one ply orientation is selected, the following equality constraint is imposed:

(3) $$\overset{Nc}{\underset{c=1}{\sum }}x_{lc}=1;\;\;\;\;x_{lc}\in \left[0;1\right],\;\;\;\;\;\forall \left(l,c\right)$$

A key point is to relax integral variables (0 or 1) as continuous ones due to the requirement of a gradient-based optimization method, especially for a density-based topology optimization approach [26]. However, the requirement of selecting only one orientation for each layer will not be satisfied if design variable $x_{lc}$ is continuous between 0 and 1. For the purpose of penalizing intermediate variables, the well-known rational approximation of material properties (RAMP) interpolation scheme [27] is employed here to push the continuum variables towards the discrete 0–1 design variables. The RAMP interpolation scheme is expressed as

(4) $$E_{el}\left(x_{lc},\rho_{el}\right)=E_{0}+\frac{\rho_{el}}{1+q\left(1-\rho_{el}\right)}\overset{Nc}{\underset{c=1}{\sum }}\frac{x_{lc}}{1+p\left(1-x_{lc}\right)}\left(E_{c}-E_{0}\right)$$

where p and q are the penalization parameters. In order to ensure that only one ply orientation is selected in each layer, the following equality constraint is imposed [28]:

(5) $$\overset{Nc}{\underset{c=1}{\sum }}x_{lc}=1,\;\;\;\;x_{lc}\in \left[0,1\right],\;\;\;\;\;\forall \left(l,c\right)$$

In the conventional DMTO method, different candidate materials (c) refers to different fiber orientations. In this study, the conventional DMTO was extended to include metal material for topology optimization of the metal reinforcement plate. Therefore, metal material was regarded as another candidate material besides the layer orientations. For the laminated composite material, the global stiffness matrix can be calculated as follows:

(6) $$K=\overset{Ne}{\underset{e=1}{\sum }}\overset{Nl}{\underset{l=1}{\sum }}\int \;B_{el}^{T}E_{el}B_{el}dv_{el}$$

where K is the global stiffness matrix and $B_{el}$ is the strain-displacement matrix of element e in layer l. $v_{el}$ is the volume of the *l*th layer in the *e*th element.

### 2.2. Manufacturing Constraints

**Figure 1:**
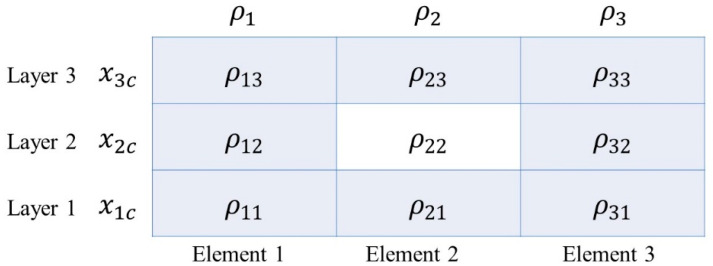
A three-layer laminate with the thickness topology variable $\rho_{el}$, through-the-thickness variable $\rho_{e}$ and ply orientation variable $x_{lc}$.

Gersborg and Andreasen originally applied the explicit parameterization for casting constraints in topology optimization of castable designs [29]. The first manufacturing constraint in this study is to prevent intermediate void. It is well known that the thickness topology variables are independent from each other, so an intermediate void structure tends to be obtained after the optimization if there are no manufacturing constraints. Taking a three-layer laminate in Figure 1 as an example, if $\rho_{22}=0$ and the elements around the central element $\rho_{12}=\rho_{21}=\rho_{32}=\rho_{23}=1$, then the intermediate void will present, which will have a negative impact on the structural performance and should be prevented.

In order to prevent the intermediate void, a Heaviside-like function is employed here [30,31]:

(7) $$\rho_{el}=1-\frac{\tanh\left(\gamma \rho_{e}\right)+\tanh\left(\gamma \left(s\left(l\right)-\rho_{e}\right)\right)}{\tanh\left(\gamma \rho_{e}\right)+\tanh\left(\gamma \left(1-\rho_{e}\right)\right)}$$

where γ is the penalization factor and *s*(*l*) is the normalized coordinate system in the thickness direction:

(8) $$s(l)=\frac{l-1}{Nl}+\frac{1}{2Nl}$$

By introducing this function, the following relation in element e can be ensured and the intermediate void will not present:

(9) $$\rho_{el}\geq \rho_{e\left(l+1\right)}$$

The second manufacturing constraint is the contiguity constraint. It aims to avoid too many adjacent layers converging to the same orientation. When the total number of adjacent layers with same orientation is too large, there is a high probability of matrix cracking, so imposing the contiguity constraint is highly necessary. In this paper, the largest number of adjacent laminate layers with same orientation was set to be 2 as follows:

(10) $$\{\begin{array}{c} \begin{array}{c} x_{lic}+x_{ljc}+x_{lkc}\leq 2\\ i=1,2,\ldots ,n^{l}-2 \end{array}\\ \begin{array}{c} j=i+1\\ k=j+1 \end{array} \end{array}$$

The last manufacturing constraint is the symmetrical layers. Nowadays, there are many ways to manufacture CFRP composite structures, such as vacuum assisted resin transfer molding (VARTM), prepregs, heat-pressing forming and so on. Some techniques may result in undesired warping and deformation due to the residual stress during manufacturing. In this case, the constraint of symmetrical layers should be applied during the optimization process to avoid failed structures. This type of constraint can be illustrated as

(11) $$\{\begin{array}{c} x_{lc}=x_{sc}\\ l+s=N^{l}+1 \end{array}$$

### 2.3. Filtering Technique

Mesh-dependence and checkerboard patterns are the common issues in classic density-based topology optimization [32]. In order to obtain a topology structure with clear material boundaries, an appropriate filtering technique needs to be applied for design variables or topological sensitivities of the original problem.

In this work, the density-based filtering technique was adopted for the design variables to avoid the checkerboard patterns after topology optimization [33]. For variable $\rho_{e}$ along the thickness direction, the new thickness variable ${\bar{\rho }}_{e}$ can be calculated as

(12) $${\bar{\rho }}_{e}=\frac{\sum_{g\in N_{e,d}}\omega \left(X_{g}\right)v_{g}\rho_{g}}{\sum_{g\in N_{e,d}}\omega \left(X_{g}\right)v_{g}}$$

where $N_{e,d}$ represents a set of elements that are within the filter radius of element e; $v_{g}$ is the volume of element g; and $\omega \left(X_{g}\right)$ is a weighted function that can be calculated as

(13) $$\omega \left(X_{g}\right)=r-\left|X_{g}-X_{e}\right|$$

where r is the filter radius and $X_{g}$ and $X_{e}$ denote the central coordinates of element g and the smoothed element e, respectively. In this study, the filter radius was set to be 10 mm for the battery-hanging structure to ensure the continuity of the material.

### 2.4. Design Sensitivity Analysis

For the gradient-based DMTO method, sensitivity analysis is essential because gradient information needs to be provided to the mathematical programming algorithm so as to solve for an optimum solution. If f represents both the objective function and constraint functions, then its sensitivity with respect to $\rho_{e}$ can be derived according to the chain rule as follows:

(14) $$\frac{\partial f}{\partial \rho_{e}}=\sum_{i\in N_{e,d}}\overset{n^{l}}{\underset{l=1}{\sum }}\frac{\partial f}{\partial \rho_{il}}\frac{\partial \rho_{il}}{\partial {\bar{\rho }}_{i}}\frac{\partial {\bar{\rho }}_{i}}{\partial \rho_{e}}$$

where $\frac{\partial {\bar{\rho }}_{i}}{\partial \rho_{e}}$ can be calculated as

(15) $$\frac{\partial {\bar{\rho }}_{i}}{\partial \rho_{e}}=\frac{\omega \left(X_{e}\right)v_{e}}{\sum_{i\in N_{e,d}}\omega \left(X_{i}\right)v_{i}}$$

where $v_{e}$ and $v_{i}$ are the volumes of element e and element i, respectively.

The sensitivity of thickness variable $\rho_{il}$ with respect to the new thickness variable ${\bar{\rho }}_{i}$ as defined in (16) can be derived as [5]

(16) $$\frac{\partial \rho_{il}}{\partial {\bar{\rho }}_{i}}=-\frac{{ℑ}_{1}-{ℑ}_{2}}{{ℑ}_{3}+{ℑ}_{4}}+\left({ℑ}_{3}+{ℑ}_{5}\right)\times \frac{{ℑ}_{1}-{ℑ}_{6}}{{\left({ℑ}_{3}+{ℑ}_{4}\right)}^{2}}$$

(17) $$\left\{ \begin{array}{c} {ℑ}_{1}=(1-{\tanh(\beta \bar{\rho }i)}^{2})\beta \\ {ℑ}_{2}=(1-{\tanh(\beta \left(s(l)-{\bar{\rho }}_{i}\right))}^{2})\beta \\ {ℑ}_{3}=\tanh(\beta \bar{\rho }i)\\ {ℑ}_{4}=\tanh(\beta \left(1-{\bar{\rho }}_{i}\right))\\ {ℑ}_{5}=\tanh(\beta \left(s(l)-{\bar{\rho }}_{i}\right))\\ {ℑ}_{6}=(1-{\tanh(\beta \left(1-{\bar{\rho }}_{i}\right))}^{2})\beta \end{array}\right.$$

If the thickness variable $\rho_{e}$ and the orientation variable $x_{lc}$ are collectively referred to as ξ, and the local displacement (u) at the loading point of the structure is regarded as the objective function, then the sensitivity of objective local displacement u with respect to design variable ξ can be derived according to the adjoint method [33] as

(18) $$\frac{\partial u}{\partial \xi }=-\lambda^{T}\frac{\partial K}{\partial \xi }u$$

where λ is the column vector with the unit value at the degree of freedom for the corresponding local displacement of interest.

## 3. Optimization of a CFRP-Laminated Battery-Hanging Structure

### 3.1. Finite Element Model

The battery-hanging structures on the floor are key components for electric vehicles because they are used to connect the floor and the battery pack. It is well known that the battery pack is of high weight and usually subjected to harsh environmental conditions, so the battery-hanging structure must have high strength and stiffness, with lighter weight being preferred. Therefore, CFRP material was adopted here due to its superior performance. The finite element (FE) model for the CFRP battery-hanging beam of an electric vehicle was developed in the commercial code ABAQUS. The equivalent single element [34,35] was adopted to model the CFRP-laminated components, in which multiple layers in a shell element were regarded as a single layer to reduce the computational cost. To calculate the stiffness matrix in each layer, Simpson-based integration technique was employed. More details of the equivalent single element and Simpson-based integration technique can be found in [4].

**Figure 2:**
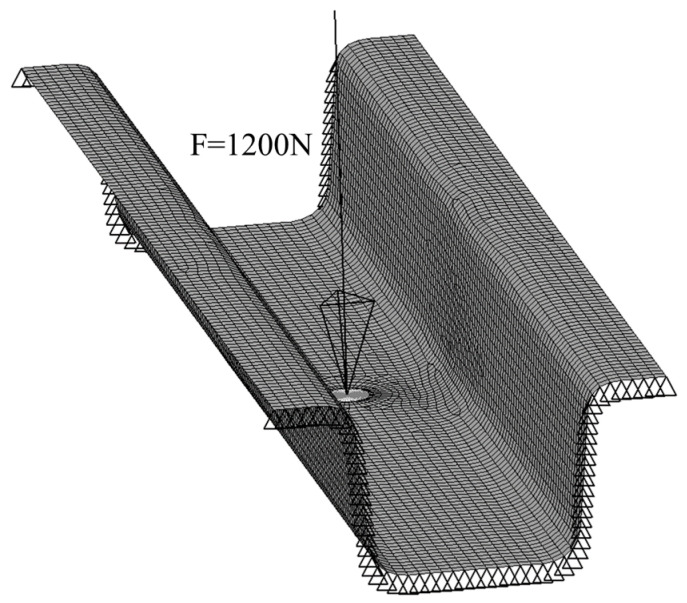
Loading and boundary condition of the finite element model.

In this model, there are several hanging beams under the floor; after distributing the weight of the battery pack to each hanging beam, the average static load applied on a single hanging beam is 200 N. When 5 times the gravity acceleration is taken as the safety factor of the battery-hanging beam, then 6 times the gravitational force should be applied to the bolt in the center of the battery-hanging beam, so 1200 N force will be applied in the vertical direction. In fact, the floor does not bear the main load; instead, the floor girder and battery-hanging beam are the main load-bearing structures. The hanging beam mainly relies on the connection with the floor girder to transmit loads. Therefore, both sides of the battery-hanging beam are fully clamped in the finite element model, as shown in Figure 2.

Four candidate ply orientations (−45°, 0°, 45°, 90°) were selected for the optimization problem for easy fabrication of CFRP. The battery-hanging beam is the connection structure between the floor and battery packs, and they are usually connected by bolts. In order to prevent the composite battery-hanging beam from failing at the position of the bolts, another choice of metal reinforcement plate was provided as a candidate material on the outermost layer of the structure, i.e., the location between the screw nut and the laminated battery-hanging beam. This section describes the simultaneous topology optimization of the metal plate and CFRP beam. Moreover, thickness topology optimization and orientation optimization of CFRP material were also conducted at the same time. Apart from the four CFRP orientations on the outermost layer, the metal plate was considered as the fifth candidate material. The material parameters for CFRP composites and metal (Q235) are summarized in Table 1 and Table 2 [36], respectively.

### 3.2. Design Optimization of the Battery-Hanging Structure

In the initial design, the battery-hanging structure comprised 11 CFPR layers, and the metal plate was also able to be selected as a candidate material in layer 12. Therefore, there were five candidates in total to be selected for layer 12, i.e., ply orientations of −45°, 0°, 45° and 90° CFRP and metal material. A value of 0.25 was assigned for initial orientation variables from layer 1 to layer 11. In layer 12, the initial value of orientation variables was 0.2 because metal material was also regarded as a candidate material. The initial values for all of the thickness variables were set to be 0.5.

In the definition of the optimization problem, the objective was to minimize the local displacement at the loading point of the battery-hanging beam while reducing the structural mass to meet the weight requirement. Accordingly, the design problem can be formulated as

(19) $$\left\{ \begin{array}{c} \mathrm{minimize}\;\;\;u_{0}\left(x_{lc},\rho_{e}\right)\\ \mathrm{subject}\;\mathrm{to}\;M\leq \eta M^{*}\\ \;\;\;\;\overset{4}{\underset{c=1}{\sum }}x_{lc}=1,l=1,2,\ldots ,n^{l}-1\\ \overset{5}{\underset{c=1}{\sum }}x_{lc}=1,l=n^{l}\\ \overset{t+2}{\underset{l=t}{\sum }}x_{lc}\leq 2\\ x_{lc}\in \left[0,1\right]\\ \rho_{e}\in \left[0.3125,1\right] \end{array}\right.$$

where $u_{0}$ is the vertical displacement at the location of the load, M is the total mass of the structure at the current iteration and ***η*** is the mass fraction which is set to be 50% of the mass $M^{*}$ of the initial fully-popularized CFRP design. The minimum of thickness variable $\rho_{e}$ is set to be 0.3125 for keeping the boundary shape of the structure, as in Wu et al.’s work [5].

The method of moving asymptotes (MMA) [37,38] was employed here to search for the optimum solution of the optimization problem defined in Equation (19). The convergence criterion is that the maximum differences between the design variables in last two consecutive iterations are less than a given tolerance, expressed as

(20) $${∥\zeta_{\delta }-\zeta_{\delta -1}∥}_{\infty }\leq \varsigma$$

where $\zeta_{\delta }$ denotes the vector of design variables at the δth iteration and ς (=0.001 here) is the prescribed convergence tolerance.

**Figure 3:**
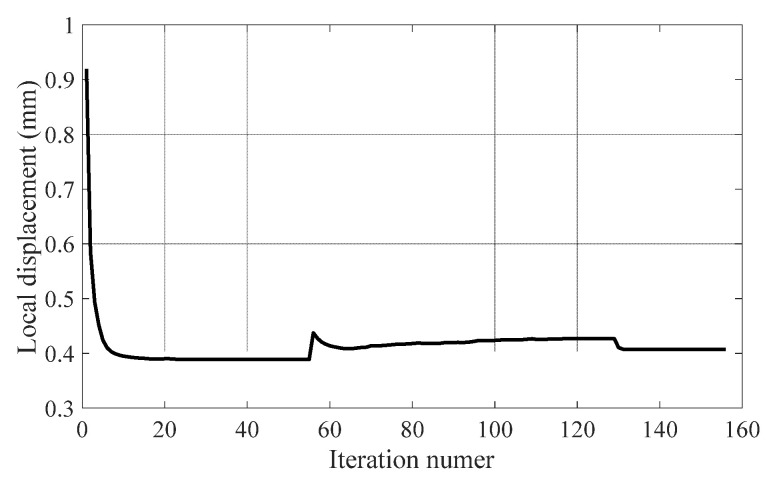
Iteration history for topology optimization of battery-hanging structure.

The iteration history for topology optimization of the battery-hanging structure composed of CFRP and metal material is plotted in Figure 3. The set of the penalization parameters (p,q,γ) would be gradually set larger if convergence was achieved or the current iteration number was larger than 1000. It can be seen that the optimization process converged well in less than 160 iterations. It should be noted that it oscillates in iterations 56 and 130 because the penalization parameters were increased suddenly when convergence was achieved in previous iterations so as to push the design variables towards discrete directions.

**Figure 4:**
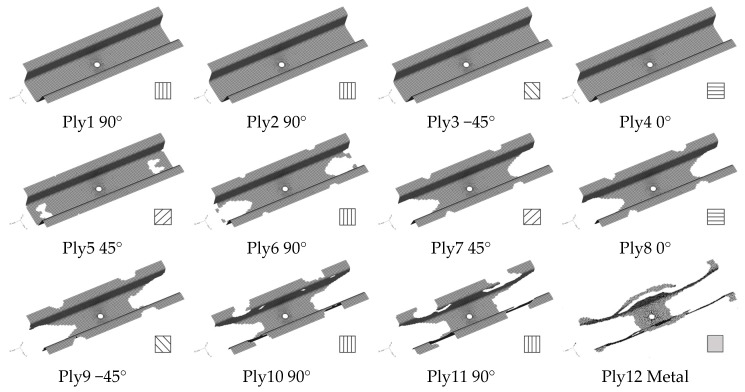
Optimized layout of each CFRP layer of the battery-hanging beam.

**Figure 5:**
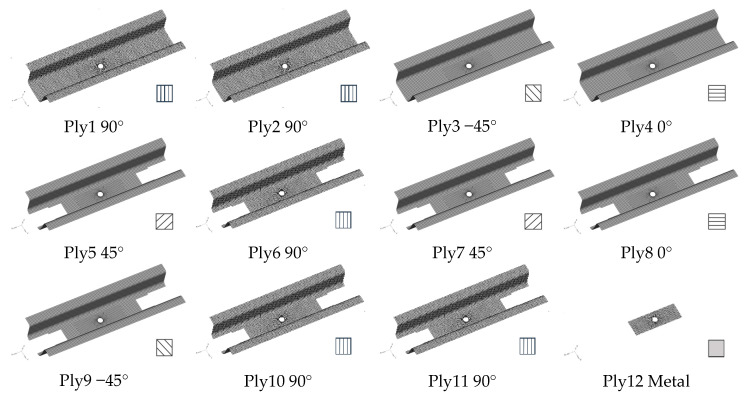
Smoothed layout of each layer after postprocessing.

The layout of each layer of the battery-hanging beam after the topology optimization is shown in Figure 4. It can be seen that the first four layers are full of CFRP material. There are some voids presented from ply 5 to ply 11, but the layout of CFRP material tapers gradually because the intermediate void can be prevented effectively due to the manufacturing constraints. Obviously, metal material was finally selected from the five candidates in layer 12 to obtain an optimum solution. However, it is difficult to manufacture without postprocessing of the optimization results, so it is necessary to smooth out the layouts from ply 5 to ply 12. Figure 5 shows the final thickness distribution of the battery-hanging beam after postprocessing for prototyping.

### 3.3. Comparison between Original Steel Structure and the Optimized Structure

**Figure 6:**
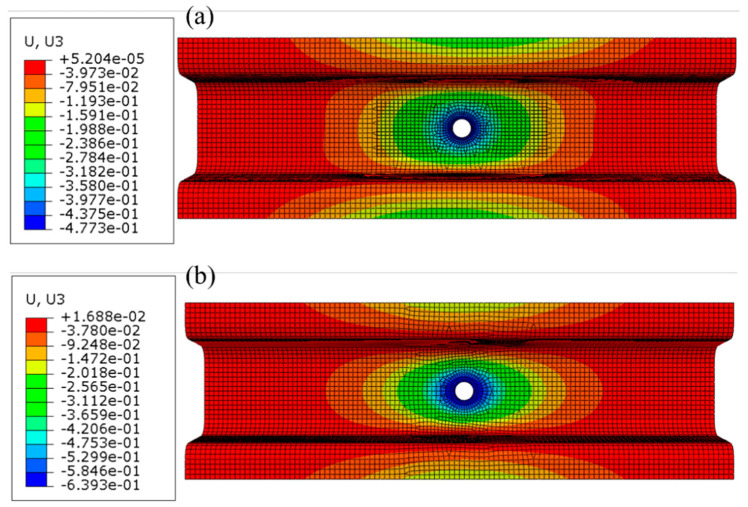
Deformation contour: (**a**) deformation of CFRP structure after postprocessing; (**b**) deformation of original steel structure.

The thickness of the original Q235 steel battery-hanging structure is 1.2 mm and the total mass is 0.610 kg. To compare the optimized structure with the original steel one, the displacement diagram of the steel structure is shown in Figure 6b; obviously, the maximum displacement is 0.64 mm at the location of the structural center. After substituting metal material with the carbon fiber reinforcement plastic material, the total mass was reduced to 0.401 kg without sacrificing mechanical performance, as shown by the maximum displacement being only 0.48 mm (presented in Figure 6a). It should be noted that the structure in Figure 6a is the one after postprocessing in consideration of ease of further manufacturing. Therefore, the total mass of the CFRP battery-hanging structure was reduced by 34.3% and the maximum local displacement was reduced by 25.3% when compared with the steel counterpart, which means the stiffness of the CFRP-laminated structure was enhanced significantly with less mass of the total structure.

## 4. Prototype and Experimental Validation

The optimized structure after postprocessing needed to be validated to determine the appropriateness and precision of the design results. Therefore, manufacturing and experimental testing were conducted, and the displacement at the location of load obtained from the test was compared with the deformation in finite element analysis. Prepregs made of unidirectional fibers were used for manufacturing. These pre-impregnated carbon fibers in which the epoxy resin already existed needed to be processed with the hot pressing technique to produce the CFRP prototype. The carbon fiber was T700 UD/epoxy resin prepreg, and the concave die and punch die were made of steel.

**Figure 7:**
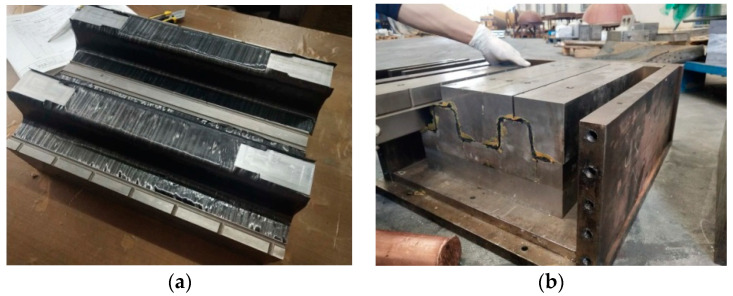
CFRP laminates on the molds and the closed molds: (**a**) laying up the prepreg; (**b**) closing the molds.

**Figure 8:**
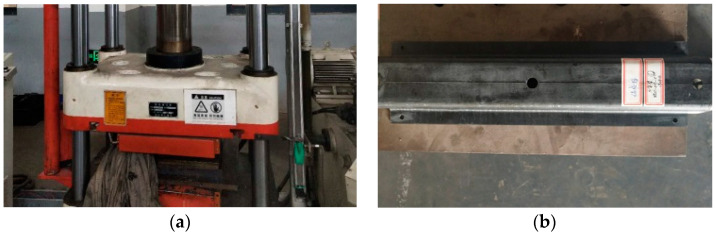
Heat-pressing forming of the prototype: (**a**) heating and pressing the structure; (**b**) the manufactured prototype.

Firstly, all possible contaminants on the surfaces of the molds were wiped with chemical cleaner. The release agent was applied on the molds to prevent the CFRP laminates from sticking to the surfaces. CFRP laminates were laid on the molds layer by layer, and the sequence of the layer orientations was according to the optimization results, as shown in Figure 7a. After placing the concave die onto the punch die in a frame, some wedge blocks were inserted in the gap between the frame and the molds because the female die and the punch needed be compacted well, as displayed in Figure 7b. Finally, the frame with the CFRP structure inside was heated and pressed on the four-column hydraulic machine, as shown in Figure 8a; the final prototype is presented in Figure 8b. In order to ensure good repeatability of the subsequent testing, three samples were manufactured.

**Figure 9:**
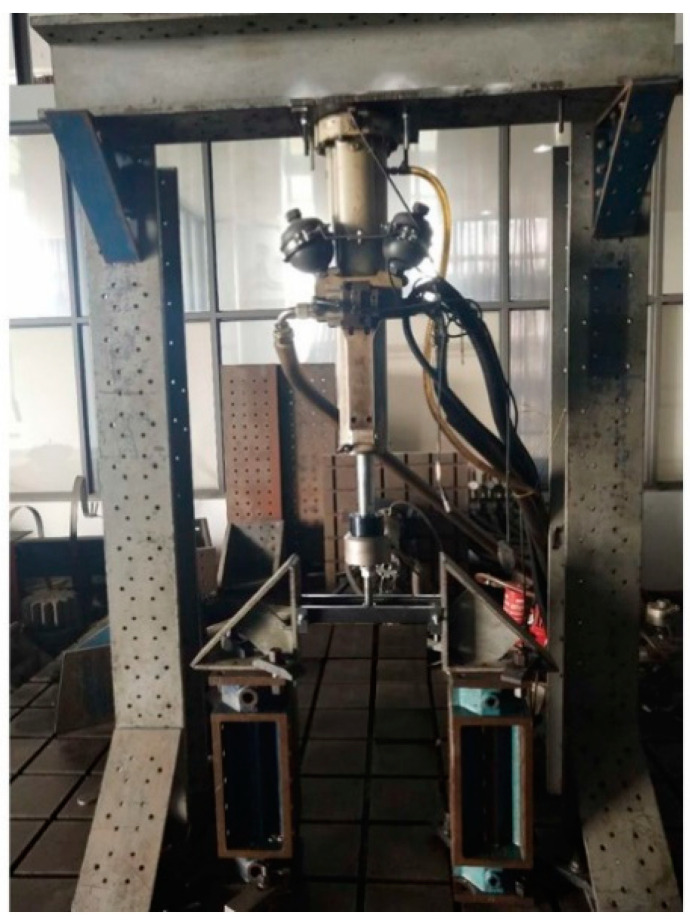
Experimental setup for the tests of the optimized battery-hanging beam.

**Figure 10:**
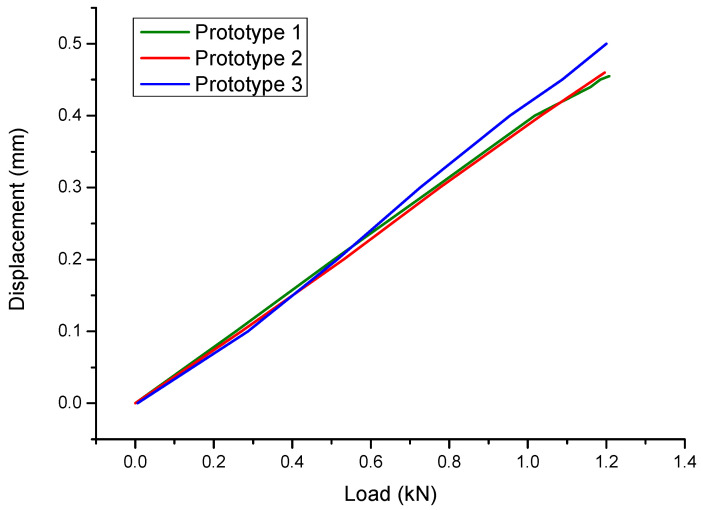
Load–displacement diagram of the experimental test.

To test whether the finite model for the optimal design was correct and accurate, the experimental tests of the prototyped battery-hanging structure were conducted, as shown in Figure 9. In the experimental tests of the three samples, the displacements at the loading point under specific load were measured. Figure 10 gives the relationship between the load and the displacement of the three samples, and Table 3 compares the results from the experimental tests and numerical simulation. Obviously, the repeatability of the test was acceptable and the FE simulation was accurate enough, which demonstrates that the topology optimization of the CFRP-laminated battery-hanging structure is effective.

## 5. Conclusions

This study extended the DMTO method to conduct topology optimization of a carbon fiber reinforced plastic laminated battery-hanging structure together with a metal reinforcement plate. Considering the actual manufacturing process, the constraints of intermediate void, largest number of adjacent laminate layers with same orientation and symmetrical layers were applied in the topology optimization. The performance of the optimized structure was compared with the original steel counterpart, revealing that a reduction of 34.3% of the total mass for the CFRP battery-hanging structure was achieved without sacrificing mechanical performance. Specifically, the maximum local displacement was reduced by 25.3% when compared with the original steel structure, which implies that the stiffness was improved significantly with less weight of the total structure. Finally, the optimal design was prototyped and tested experimentally to validate the effectiveness of the proposed approach.

## Figures and Tables

**Table 1:** Material parameters of carbon-fiber-reinforced plastic (CFRP).

Symbol	Parameters for CFRP Material	Value
$t_{c}$	Ply thickness for CFRP	0.2 mm
$E_{1}$	Longitudinal Young’s modulus	90 GPa
$E_{2}$	Transverse Young’s modulus	6.5 GPa
$G_{12}$	Shear modulus	3.5 GPa
$\rho_{c}$	Density for CFRP	1900 kg/m^3^
$X_{t}$	Longitudinal tensile strength	1800 MPa
$X_{c}$	Longitudinal compressive strength	1100 MPa
$Y_{t}$	Transverse tensile strength	30 MPa
$Y_{c}$	Transverse compressive strength	144 MPa
$S_{12}$	Shear strength	60 MPa
$v_{12}$	Poisson’s ratio	0.3

**Table 2:** Material parameters of metal on the outermost layer.

Symbol	Parameter for Metal Material	Value
$E_{m}$	Young’s modulus	210 GPa
$t_{m}$	Thickness	1.2 mm
$v_{m12}$	Poisson’s ratio	0.3
$\rho_{m}$	Density	7900 kg/m^3^

**Table 3:** Comparison between the FE simulation and experimental tests of the optimized structure.

	Protype 1	Protype 2	Protype 3	Mean	FEA	Error
Displacement (mm)	0.44	0.45	0.49	0.460	0.477	3.70%
